# Achieving Precision Mental Health through Effective Assessment, Monitoring, and Feedback Processes: Introduction to the Special Issue

**DOI:** 10.1007/s10488-016-0718-5

**Published:** 2016-02-18

**Authors:** Leonard Bickman, Aaron R. Lyon, Miranda Wolpert

**Affiliations:** Vanderbilt University, Nashville, TN USA; Department of Psychiatry and Behavioral Sciences, University of Washington, 6200 NE 74th St., Suite 100, Seattle, WA 98115 USA; Evidence Based Practice Unit, UCL and the Anna Freud Centre, 12 Maresfield Gardens, London, NW3 5SU UK

## Personalization and Precision: A New Paradigm

There is a sense of excitement and change occurring in mainstream medicine. President Obama, in his State of the Union address on January 30, 2015, announced a national Precision Medicine Initiative (The White House 2015). More recently, the United Kingdom’s government innovation agency started a Precision Medicine Catapult designed to enhance the development of precision medicine in the UK (Precision Medicine Catapult 2015). Precision medicine is defined by the National Research Council as “the tailoring of medical treatment to the individual characteristics of each patient” (National Research Council (US) Committee on A Framework for Developing a New Taxonomy of Disease 2011). This builds on an increasing interest in personalized medicine and, indeed, the terms “precision medicine” and “personalized medicine” are sometimes used interchangeably (Avitabile 2015). Common to both is an emphasis on tailoring treatment to individual needs and, increasingly, on the role of technology to support that goal (Carney 2014; Sacchi et al. 2015).

Although much of the focus of medicine to date has been on biomarkers and genetics (McCarty et al. 2011), the concept is not limited to those factors. Just as critical, but less widely elaborated, are psychosocial variables that also fit under the umbrella of precision and personalized medicine. Increasing discussion has also focused on the relevance of precision medicine to mental health. Thomas Insel, a former Director of the National Institute of Mental Health (NIMH), has argued that the basic tenets of precision medicine are reflected in the NIMH Research Domain Criteria (RDoC) project, “which aims to develop more precise diagnostic categories based on biological, psychological, and socio-cultural variables” (Insel 2015). He adds that

“…precision medicine for mental disorders will not come from a single genomic glitch. Rather, like many other areas of medicine, many genes each contribute only a small amount of vulnerability as part of an overall risk profile that includes life experiences, neurodevelopment, and social and cultural factors. RDoC assumes that we will need many kinds of data to reach precision, more like triangulating to find your position on a map. These data will draw from many sources, including symptoms, genotype, physiology, cognitive assessment, family dynamics, environmental exposures, and cultural background.”

The NIMH’s emphasis on including a wide variety of assessment data in the pursuit of precision recognizes that mental health must move beyond genetic factors as the sole focus of RDoC-facilitated precision.

## Precision Mental Health: Definitions and Requirements

For precision medicine to become a reality in mental health, it is necessary to have precise assessment, monitoring, and feedback information. We define *precision mental health* as an approach to prevention and intervention that focuses on obtaining an accurate understanding of the needs, preferences, and prognostic possibilities for any given individual, based on close attention to initial assessment, ongoing monitoring, and individualized feedback information, and which tailors interventions and support accordingly in line with the most up-to-date scientific evidence. In particular, this data-driven approach to clinical decision-making should include seven types of psychosocial data, which are described below (and summarized in Table 1):

1. *Personal data* relevant to understanding the nature of presenting problems and how they might be addressed may include description of the presenting problem and/or psychiatric diagnoses, but also consideration of other factors, including genetic, developmental, social, and cultural variables. We anticipate that this will go beyond more than just symptoms, to include prominent and systematic consideration of information that may inform intervention choices, including motivation to change, personality traits, and demographics.
2. *Aims and risks data* Clarifying the focus and expected outcomes of treatment as well as any risks or likely side effects is a key issue for mental health and one that currently is all too often hazy or ill-defined. This does not mean that service users get to choose *any* aim or goal to work on and the service provider has to comply; rather, this is about capturing what has been mutually agreed as the focus for treatment to allow precision in terms of tailoring the intervention to the aim, along with any acknowledged risks, and ensuring progress toward this end. Service recipients with identical symptom profiles and case formulations often have different aims, and these aims may further diverge from those of their care provider. Precision mental health tailors activity to the specific agreed-upon aims.
3. *Service preference data* relevant to understanding patient/client choices at key decision points regarding services. Similar to aims data, service recipients with identical symptom profiles and case formulations may have divergent preferences for different interventions. In situations where the evidence for two different interventions is relatively equally balanced, then preference data are crucial to help guide intervention selection, to ensure personalization and precision (Jacob et al. 2015), and to prevent misdiagnosis of preference (Mulley et al. 2012).
4. *Intervention data* that capture aspects of the services delivered over the course of treatment, including their dose/intensity, duration, cost, and timing. This includes precision as to different interventions and aspects of interventions, and may benefit from taxonomies that are not just modality based, using the TIDieR framework to capture details of interventions (Hoffmann et al. 2014). These include the behavioral taxonomy developed by Michie et al. (2014) and the “common elements” of evidence-based treatments suggested by Chorpita et al. (2005), alongside more traditional “common factors” identified in the literature (Bickman 2005). Aspects of intervention integrity/fidelity (i.e., adherence, competence, differentiation, and relational elements; Southam-Gerow and McLeod 2013) also represent key aspects of intervention data.
5. *Progress data* relevant to understanding movement toward the intended and agreed aims of any intervention and against identified benchmarks (see #3 above). These data are collected routinely over time using within-subjects comparisons and relevant metrics as identified in #1 and #3 above.
6. *Mechanisms data* relevant to the hypothesized link between intervention and outcomes (Kazdin, 2007). These are frequently the hypothesized mediators of treatment. For example, therapeutic alliance would be included as an explanatory factor if it is not considered to be an explicit component of the intervention (see #5), but this might also include skills developed by the service recipient as part of the intervention, such as increased coping skills or social skills.
7. *Contextual data* relevant to understanding the factors that moderate or mediate outcomes, such as quality and amount of service available, or other data external to the individual or the intervention delivered (which are captured in #4 and #6). These are data about the environment in which the individual lives, in contrast to personal data (described in #1).

**Table 1:** Types of psychosocial data relevant to precision mental health

Data type	Description
Personal data	Individual-level information that may inform intervention choice/selection (e.g., demographics; diagnoses; cultural variables; motivation to change)
Aims and risks data	The focus and expected outcomes of treatment as well as potential risks
Services preference data	Client choices/selections at key decision points regarding services
Intervention data	Aspects of the services delivered over the course of treatment (e.g., intervention integrity; dose/intensity; duration; timing)
Progress data	Movement toward the intended and agreed aims of any intervention, and against identified benchmarks
Mechanisms data	The hypothesized link between intervention and outcomes. May be mediators of treatment (e.g., skills development or use, therapeutic alliance, etc.)
Contextual data	Factors external to the individual/intervention that moderate or mediate outcomes (e.g., quality and amount of service available; family functioning data)

Precision mental health can be distinguished from current “best practice” in mental health promotion and provision in the following ways. First, it involves careful, ongoing consideration of the seven data elements above over the course of any intervention. In this way, precision mental health should be “data driven” in a manner that extends well beyond the growing contemporary emphasis on client outcome tracking. Second, given the extensive data that will be required to make precision mental health a reality, our conceptualization is committed to using relevant technology to manage information and support precision in assessment monitoring and feedback. It should be acknowledged, however, that precision mental health is currently an aspirational goal and that much of the current data in mental health are largely flawed and proximate (Wolpert et al., 2014). In light of this, those seeking to support precision mental health need to take due account of the imprecision of current data sources.

## Precision Mental Health, Measurement, and Feedback in Clinical Practice

This special issue marks a step toward considering current best practice in using these data sources to support precision mental health across both the United States and the United Kingdom. Although none of the authors in the present issue have conceptualized their work in terms of precision mental health, we feel that all the contributors are working toward this end. We advocate that, as a community of researchers and practitioners, we should begin to frame the collection and use of patient-reported outcomes and other measures in terms of precision mental health. We anticipate that doing so will not only facilitate alignment between mental health and the broader healthcare agenda, but also help to overcome some terminology differences that have emerged in the areas of outcome monitoring and feedback, which we would like to redress.

In particular, there is a plethora of terms used across the literature to refer to various components of precision mental health services. These include Measurement-Based Care (MBC) (Scott and Lewis 2015), Outcome-Informed Therapy (Duncan et al. 2011), Feedback Informed Therapy (FIT) (Miller et al. 2015), Routine Outcome Monitoring (ROM) (Carlier et al. 2012), and Measurement Feedback Systems (MFS) (Bickman 2008). Among these, ROM and MFS are the two most common shorthand terms that have come to be used differently across the United States and United Kingdom to refer to the varied elements of the assessment, monitoring, and feedback process. The former emphasizes the importance of collecting data that inform an understanding of outcomes—with a focus particularly on #1–3 and 5 above—and is widely used in the United Kingdom. The latter emphasizes the use of systems to provide feedback from those accessing services, which also focuses on data related to #1–3 and 5 above, but has additionally paid more attention to other relevant data on a routine basis, including mechanism data (#6 above), and consideration of the nature of interventions (#4 above). This includes natural language descriptions of the content of treatment above (Kelly et al. this issue) and specific evidence-based intervention components (Chorpita et al. this issue). In practice, the terms are often used interchangeably, and those promoting ROM and MFS approaches share a common commitment to systematically capturing data and supporting clinicians to make use of all the elements listed above. Regardless of the terminology, this is a revolutionary perspective given that traditional mental health intervention does not involve any systematic data collection or considerations of outcomes from the user perspective (Garland et al. 2003; Hatfield and Ogles 2004). Moreover, these are universal approaches to improving outcomes that can be used regardless of the type of treatment or characteristics of the client or clinician. We would advocate that they be increasingly subsumed under the term precision mental health.

## Precision Mental Health: Challenges and Opportunities

### Relevant Data Components

We anticipate that the advancement of precision mental health will require greater use of data sources not yet fully tapped by current approaches to mental health symptom assessment, such as educational- or employment-related functioning, cognitive and neurological testing, and other bio-social indicators (relevant to #1, 6, and 7 above). There is no conceptual reason why these data elements cannot be increasingly integrated into feedback systems, particularly as systems move to be largely digital and cloud-based with rapid real-time reporting possible (Lyon et al. 2016). However, since many of the measures developed in this area are laboratory-derived, a translational process might be necessary to make them feasible in the real world. For example, Bickman and colleagues developed a battery of measures that are designed for use in real-world settings where time is short (Bickman and Athay 2012).

Moreover, precision mental health provides an opportunity for the field to move beyond traditional self-report data. Almost all the data currently collected are based on clients’ or others’ completion of questionnaires. Although such an approach provides critical information about clients’ perceptions of their own difficulties, this mono-method dependency is problematic. While we are aware that we still have much work to do to integrate and understand self-report data (De Los Reyes 2011), we are missing new and rapidly emerging sources of information. For instance, Torous and Baker (2016), as well as many others, have noted that the new technologies based on smartphones and wearable sensors offer access to data and events that are not possible with electronic or paper-based questionnaires completed in the office or clinic. Although there are numerous complex issues that need to be resolved with the use of these new technologies (e.g., privacy, security, validity), there is significant potential to transform what we know about mental health and mental health services.

Among the data sources captured in the list presented above, information about the intervention itself (#4 above) is particularly underdeveloped. Physical medicine is going through a major cultural shift from the practice of medicine as an art to medicine that is evidence based and follows guidelines and standards. However, this has not been a simple journey, and some of the problems encountered may be remedied by an emphasis on precision medicine (Greenhalgh et al. 2014). Although many evidence-based treatments exist in mental health, research indicates that these are not yet part of the mainstream clinical culture (Becker et al. 2013). Moreover, there is currently little incentive for providers to use these treatments and monitor their fidelity. Thus, most care is described using the imprecise—and typically heterogeneous—term “treatment as usual”. Many of the feedback studies to date have introduced feedback practices into that “treatment as usual” context, which may not be optimal. This lack of precision in describing treatment is a handicap for feedback systems, because is it unclear not only what data to relay, but also what actions the clinician should take based on the feedback. The use of frameworks to identify intervention components (e.g., Chorpita et al. 2005; Michie et al. 2014) should continue to be advanced, but they are not yet widely embedded in practice, as will be noted in many of the contributions to this special issue.

### Building Precision Mental Health Databases

The mental health field lacks high-quality, large databases that include linked data from #1 to #7 above. Databases currently available to form the basis for precision medicine are likely to be drawn from three sources: clinical trials, routine care, and cohort studies. While we could find no systematic data on the sizes of clinical trials, ClinicalTrials.gov, as of December 2015, lists 192,475 trials, 7366 (3.8 %) of which deal with some aspect of mental health. Most of these include some elements of #1–7, but not all. Furthermore, many will be limited in the populations covered. Cohort studies including those developed by groups of volunteers are a potentially useful source of data (Precision Medicine Initiative Working Group, 2015), but the mental health aspects of such databases are typically limited. For the foreseeable future, routine care is likely to be the key source of data for pursuing precision mental health. However, these datasets are likely to be highly flawed and incomplete, suffering from the challenges common to administrative datasets (e.g., missingness, inadequate specification) and exacerbated by the fact that, in mental health, we will have to depend on typical community-based treatment. Significant sources of data for health care are hospital data systems and laboratory test results. Hospitals and laboratories have a long history of collecting and maintaining relatively high-quality data, but outpatient mental health services often do not share this tradition. Furthermore, most existing data systems are not designed to “talk” to each other. This interoperability problem exists in physical medicine, but there are financial incentives for providers to develop such systems (e.g., Blumenthal and Tavenner 2010). Moreover, there are large investments being made by governments to create solutions.

Presently, ROM and MFS are in the forefront of developing technologies suitable for mental health to obtain the needed data. However, given the lack of similar incentives and financial resources, and the lack of standardized and widespread measurement, progress will be slow. The quality of mental health data from routinely collected data sources is therefore likely to remain a problem for some time to come. Many of the papers in this special issue deal with the problems inherent in collecting such data in the real world.

### Facilitating Ease of Data Capture and Use in Mental Health

One of the major challenges this field faces concerns the implementation of data capture and use in the context of under-resourced and overstretched services. In many cases, new measures must be developed because the existing measures were developed for research projects without severe time restrictions for data collection. The resources available in research settings stand in contrast to the conditions of service delivery in the real world, where assessment is often seen as “stealing” time from treatment. Furthermore, the focus on monitoring makes more relevant individualized (i.e., idiographic) assessment approaches that are typically used for intra-individual comparisons (i.e., comparing individuals with themselves over time), rather than comparing individuals with established norms from a larger population (Haynes et al. 2009; Weisz et al. 2011). Many of the articles in this issue address the issue of implementation and draw on implementation science for suggested ways forward.

### MFS and ROM Support Precision Mental Health

The current special issue contains two companion sections that showcase projects designed to support the elements of precision mental health listed above. They address some of the challenges previously identified via different technical (i.e., training, consultation, learning collaborative) and technological (i.e., digital measurement feedback systems and electronic health records) strategies. The special issue arose because of a range of work going on across the United States, United Kingdom, and elsewhere (e.g., the Netherlands) where researchers and practitioners were experiencing common challenges and concerns. Originally designed as two separate contributions, the commonalities between the groups became clear and therefore they were brought together in one issue while treating each section with its own introduction and overview. For specific information about the individual article author contributions, the reader is referred to the individual special section introductory papers. Specifically, Edbrooke-Childs, Wolpert and Deighton (this issue) have prepared a section focused on the use of patient-reported outcome measures (PROMs), which includes consideration of training and support necessary to allow for implementation. Lyon and Lewis (this issue) oversee a section that focuses on the development and implementation of digital MFS technologies explicitly designed to support ROM practice.

Papers in both sections stress that implementation and long-term sustainment of using patient-reported outcomes and other data to inform practice can be fraught with challenges, such as varying levels of organizational buy-in, long timelines, and mounting costs. Nevertheless, they also demonstrate the potential payoffs of successfully installing these innovations. Furthermore, the papers make it clear that the implementation of feedback technologies involves many of the same issues as those involved in the implementation of other evidence-based practice changes in behavioral health. Thus, they require good design and packaging to make them accessible and useable for practitioners, and to facilitate their uptake and long-term use. This may be accomplished by explicitly incorporating stakeholders and stakeholder perspectives into structured processes for the development, selection, and implementation of new innovations. Consistent with the broader implementation literature (Beidas and Kendall 2010), effective training and consultation procedures are necessary regardless of the type of innovation being implemented. Furthermore, both sections make clear the value of qualitative, quantitative, and mixed-methods approaches to (1) evaluate clinician and service recipient views toward the technological and practice changes that characterize implementation of feedback approaches, (2) tailor the practices or technologies to meet their needs, and (3) determine their effectiveness in promoting positive service outcomes. With this special issue, we hope to advance the science and practice of precision mental health by considering the capture, feedback, and use of data in community service settings, as well as the processes and strategies through which these innovations are developed, implemented, and evaluated.
